# Brain Imaging of Vesicular Monoamine Transporter Type 2 in Healthy Aging Subjects by ^18^F-FP-(+)-DTBZ PET

**DOI:** 10.1371/journal.pone.0075952

**Published:** 2013-09-30

**Authors:** Kun-Ju Lin, Yi-Hsin Weng, Chia-Ju Hsieh, Wey-Yil Lin, Shiaw-Pyng Wey, Mei-Ping Kung, Tzu-Chen Yen, Chin-Song Lu, Ing-Tsung Hsiao

**Affiliations:** 1 Department of Medical Imaging and Radiological Sciences, College of Medicine, Chang Gung University, Taoyuan, Taiwan; 2 Healthy Aging Research Center, Chang Gung University, Taoyuan, Taiwan; 3 Department of Nuclear Medicine and Molecular Imaging Center, Chang Gung Memorial Hospital, Taoyuan, Taiwan; 4 Division of Movement Disorders, Department of Neurology, Chang Gung Memorial Hospital, Taoyuan, Taiwan; 5 Neuroscience Research Center, Chang Gung Memorial Hospital, Taoyuan, Taiwan; 6 School of Medicine, Chang Gung University, Taoyuan, Taiwan; 7 Department of Radiology, University of Pennsylvania, Philadelphia, Pennsylvania, United States of America; Northwestern University Feinberg School of Medicine, United States of America

## Abstract

^18^F-FP-(+)-DTBZ is a novel PET radiotracer targeting vesicular monoamine transporter type 2 (VMAT2). The goal was to explore the image features in normal human brains with ^18^F-FP-(+)-DTBZ as a reference of molecular landmark for clinical diagnosis in Parkinson's disease (PD) and related disorders.

**Materials and Methods:**

A total of 22 healthy subjects (59.3±6.0 years old) including 7 men and 15 women were recruited for MRI and ^18^F-FP-(+)-DTBZ PET scans. A total number of 55 brain VOIs were selected for quantitation analysis. The regional specific uptake ratio (SUR) was calculated with occipital as reference from MRI-based spatially normalized ^18^F-FP-(+)-DTBZ images. Regional percentage SUR to that of anterior putamen was calculated. Average SUR images were displayed in 2D and 3D space to illustrate the image patterns. The correlation between age and regional VMAT2 uptake was also examined.

**Results:**

Visual assessment showed symmetric uptake of ^18^F-FP-(+)-DTBZ and obviously highest in striatum, followed by nucleus accumbens, hypothalamus, substantia nigra, and raphe nuclei. Quantification analysis revealed striatal VMAT2 density of anterior putamen>posterior putamen>caudate nucleus. Other subcortical regions were with moderate VMAT2 distribution (6∼51% SUR of anterior putamen), while slightly lower VMAT2 was observed in cerebellum (10.60% SUR) and much lower in neocortex (<5% SUR). No significant correlation of SUR to age was found in subcortical regions.

**Conclusion:**

Using ^18^F-FP-(+)-DTBZ PET, we showed the 2D and 3D imaging features of the VMAT2 distribution *in vivo* in healthy aging brains. The *in vivo* imaging characteristics of VMAT2 is consistent with the expression of VMAT2 in a recent autopsy study. Therefore, 3D visualization and higher image quality of ^18^F-FP-(+)-DTBZ PET imaging might potentially be a powerful biomarker in detecting VMAT2 distribution of subcortical regions, and for Parkinson's disease and related neuropsychiatric disorders involving related monoaminergic systems.

## Introduction

The type 2 vesicular monoamine transporter (VMAT2) is a protein responsible for pumping monoamine neurotransmitters, including dopamine (DA), norepinephrine (NE) and serotonin (SE), from the neuronal cytosol into synaptic vesicles. Measurement of the VMAT2 density in brain can serve as a diagnosis tool for many neuropsychiatric diseases related to monoaminergic dysfunction. Since more than 90% of VMAT2 locate in the dopamine nerve terminals [1], the *in vivo* estimation of VMAT2 density may have great help for the diagnosis and management of disorders associated with nigrostriatal degeneration, such as Parkinson's disease (PD). In previous studies, positron emission tomography (PET) imaging with the VMAT2 tracer ^11^C-dihydrotetrabenazine (^11^C-DTBZ) has been proven to be an objective marker of nigrostriatal terminal integrity [2].

A novel tracer ^18^F-9-fluoropropyl-(+)-dihydrotetrabenzazine (^18^F-FP-(+)-DTBZ) for VMAT2 imaging with a longer half-life (t_1/2_ = 110 min) has been recently developed [3]. ^18^F-FP-(+)-DTBZ PET imaging has a high sensitivity for detecting dopaminergic integrity in both healthy subjects and PD patients [4], [5], [6]. Yet the detailed imaging characteristics of VMAT2 distribution in healthy aging human brains with ^18^F-FP-(+)-DTBZ PET have not been well described.

PD is a neurodegenerative disorder characterized by a clinical spectrum of motor and non-motor disabilities. The major pathological feature is the degeneration of DA neurons in the substantia nigra (SN) and axons projecting to the striatum [7]. In addition to the nigrostriatal DA system, the mesocortical/meolimibic pathways and those involving the NE and SE systems also play important roles in the neuropsychiatric symptoms and prognosis of PD [8], [9], [10], [11]. Thus, to study the *in vivo* VMAT2 distribution in the whole brain, particularly in the extrastriatal regions, is very important for further clinical applications, and for exploration of extended diagnostic utility of the novel tracer.

Following recent advances in the resolution and sensitivity of PET, the goal of this study is to establish the imaging features of the ^18^F-FP-(+)-DTBZ for normal VMAT2 distribution, and establish a normal basis for future diagnostic comparison in VMAT2 imaging studies. In particular, the 2D (two-dimension) and 3D (three-dimension) distribution patterns of VMAT2 binding within the dopamine pathways in normal aging brain were further investigated for a better visual analysis. In addition, the quantification on VMAT2 binding was also measured and compared between different brain regions of dopaminergic systems. All the obtained imaging data were compared with the results from previous in vitro studies [12], [13], [14].

## Materials and Methods

### Subjects

A total of 22 healthy subjects including 7 men and 15 women with age 59.3±6.0 years (range: 52–71 year) were enrolled. The study protocol was approved by the Institutional Review Board of the Chang Gung Memorial Hospital and the Food and Drug Administration, Taiwan, and a written informed consent was obtained prior to all procedures for each participant. Healthy subjects had no evidence of significant neurodegenerative disease by history and neurologic testing, and no clinically significant or unstable medical or psychiatric illness, and neither any documented abnormality in the MRI of brain.

### Data Acquisition

^18^F-FP-(+)-DTBZ was prepared at the cyclotron facility of Chang Gung Memorial Hospital as described previously [15]. PET scans were performed for all subjects in a Biograph mCT PET/CT System (Siemens Medical Solutions, Malvern, PA, USA) with a 3-dimentional acquisition mode. In addition, all subjects underwent magnetic resonance imaging (MRI) on a 3T Siemens Magnetom TIM Trio scanner (Siemens Medical Solutions, Malvern, PA, USA) for screening of other diseases, obtaining structural information, generating volumes of interest (VOIs), and performing spatial normalization with PET images.

After injection of 383±14 MBq ^18^F-FP-(+)-DTBZ, a single 10-min PET scan was acquired 90 min post injection in 3D mode [5]. PET images were then reconstructed using 3D OSEM algorithm (4 iterations, 24 subsets; Gaussian filter: 2 mm; zoom: 3) with CT-based attenuation correction, and also scatter and random correction as provided by the manufacture. The reconstructed images were with a matrix size of 400×400×148 and a voxel size of 0.68×0.68×1.5 mm^3^.

### Image Analysis

All image data were processed and analyzed using PMOD image analysis software (version 3.3, PMOD Technologies Ltd, Zurich, Switzerland). The 3D visualization of the VMAT2 distribution was processed and displayed as overlaid with the corresponding nigrostriatal VOIs using the AVIZO software (version 7, VSG Company, Germany). Each PET image was co-registered to the corresponding MR image, and the individual MR image was spatially normalized to the Montreal Neurological Institute (MNI) MRI template [16]. The spatial normalization parameters were then applied to PET images to form a final, spatially normalized PET image in the MNI domain. A total number of 58 VOIs within subcortex, neocortex, cingulum, and cerebellum were defined on the MNI template for later analysis. Nineteen subcortical VOIs were delineated on the MNI MRI template manually including bilateral caudate nuclei, anterior/posterior putamen, nucleus accumbens (NAc), substantial nigra (SN), raphe nuclei, locus coeruleus, brain stem, hypothalamus, amygdala, hippocampus, parahippocampus, and thalamus. In addition, a total number of 36 VOIs of neocortex, cingulum, and cerebellum were defined on the AAL VOI template for later analysis [17] (including vermis, bilateral anterior cingulum, middle cingulum, posterior cingulum, motor area, middle frontal, inferior frontal, medial frontal, superior parietal, inferior parietal, superior temporal, middle temporal, inferior temporal, superior occipital, middle occipital, and inferior occipital regions). The specific uptake ratio (SUR) for each VOI was calculated as [(uptake in target VOI-uptake in reference VOI)/uptake in reference VOI] [18] to reflect an estimate for the specific binding in different regions. The inferior occipital cortex was applied as the reference region [5]. The mean values from bilateral subcortical, neocortical, cingulate, and cerebellar VOIs were calculated for distribution comparison. In addition, a percentage SUR was also calculated as the normalized VMAT2 levels to the structure with the highest uptake [12].

### Statistical Analysis

The difference between left and right sides regional SUR was statistically compared using the nonparametric Mann-Whitney test, and the percent difference was also calculated for left and right sides comparison. The correlation between age and VMAT2 uptake level was examined for each VOI using Spearman's correlation coefficient. P value of 0.05 was defined as the threshold of statistical significance in all tests.

## Results

**Figure 1:**
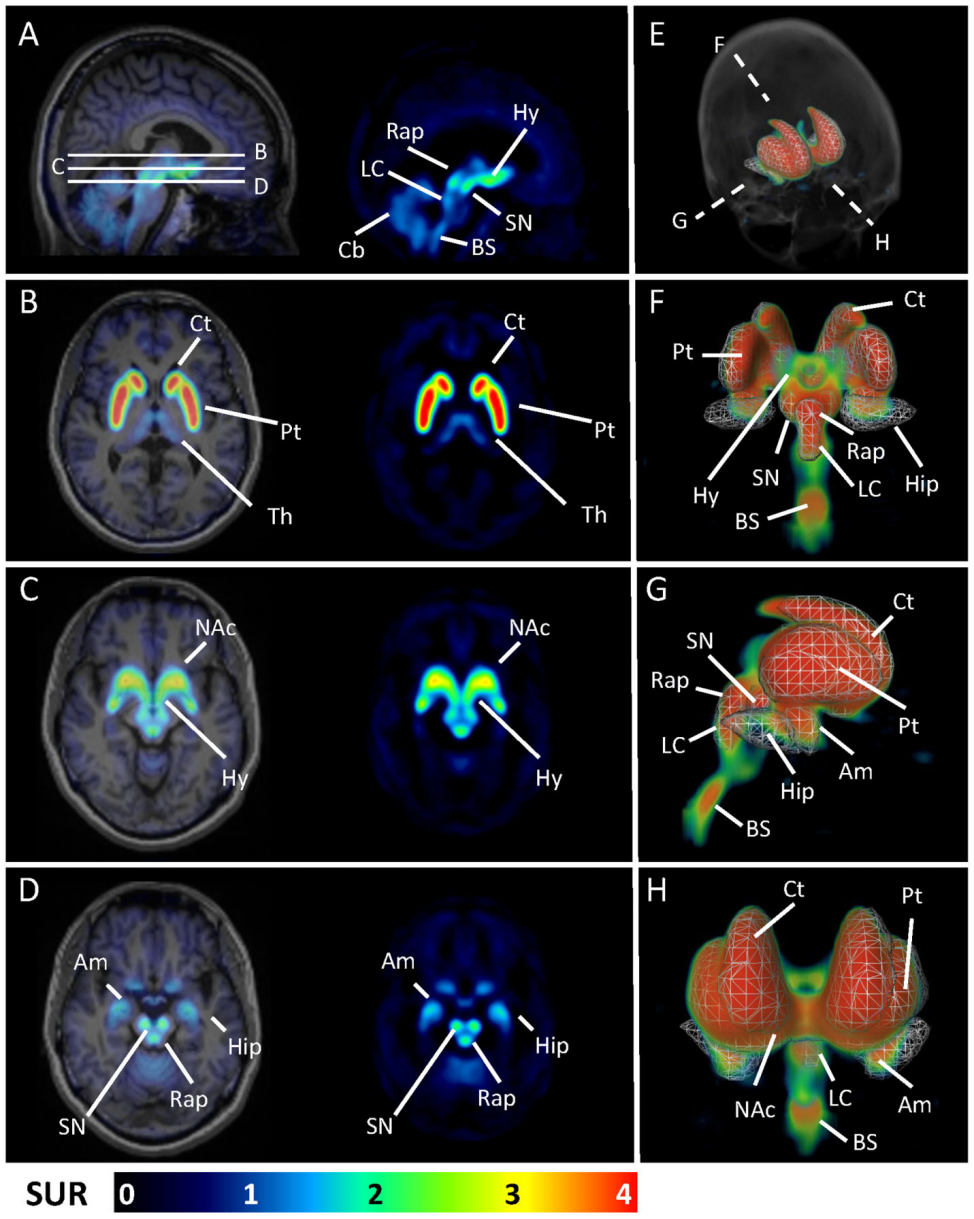
2D and 3D views of the average VMAT2 distribution in normal aging brain using ^18^F-FP(+)-DTBZ. The sagittal view of the VMAT2 distribution in the section of right substantia nigra was displayed in (A) with the three lines indicating three different 2D transverse views as illustrated in (B), (C) and (D) at the level of striatum, NAc, and brain stem, respectively. The 3D visualizations of subcortical VMAT2 binding were illustrated in (E) and also at the views of (F) posterior, (G) right lateral, and (H) anterior with corresponding VOI.

The average ^18^F-FP-(+)-DTBZ 2D and 3D SUR images from 22 healthy subjects are illustrated in Figure 1. The sagittal view of the PET images in the section of right SN was displayed in Figure 1A. The uptake of ^18^F-FP-(+)-DTBZ was obviously high in hypothalamus, SN, and raphe nuclei, and slightly increased in cerebellum. There was almost no visible distribution of ^18^F-FP-(+)-DTBZ in the cortex. In Figure 1A, the three lines indicate three different 2D transverse views as illustrated in Figure 1B, 1C and 1D at the level of striatum, NAc, and brain stem, respectively. The uptake of ^18^F-FP-(+)-DTBZ was symmetric and obviously the highest in striatum, followed by NAc, hypothalamus, SN, raphe nuclei, locus coeruleus, brain stem, amygdala and hippocampus. The caudate and putamen were well defined and could be easily differentiated from each other in the PET image without coregistration of MRI. The VMAT2 density was much lower in all cortical regions, whereas the uptake of ^18^F-FP-(+)-DTBZ in cerebellum was higher and similar to those of hippocampus, thalamus and amygdala. Furthermore, we illustrated the VMAT2 density as a 3-D picture for the major VMAT2 systems (Figure 1E) at the posterior (Figure 1F), right lateral (Figure 1G), and anterior (Figure 1H) views. In addition, a movie is shown in the supporting Figure S1 to illustrate the 3D visualization of subcortical VMAT2 binding in different angles. This revealed an integral and visible functional anatomy of dopaminergic, serotoninergic, and norepinephrinergic innervations with clear spatial correlation in 3D space.

Table 1 shows the mean SUR from 58 VOIs divided into 4 groups including subcortical, cerebellum, cingulate, and neocortex. There was no significant SUR difference between left and right sides of each region (p value<0.05). The average and standard deviation of SUR in all regions were shown in the first column of Table 1. The uptakes of ^18^F-FP-(+)-DTBZ were the highest in the anterior putamen (SUR = 2.78), followed by posterior putamen (2.68) and caudate (2.27). For each region, the percentage of regional SUR to that of the anterior putamen was also calculated and shown in the second column of Table 1. The VMAT2 density in the SN (SUR = 1.08) was about 38.75% of striatal SUR. Other high uptake ratios in the subcortical regions were observed in NAc (1.43), hypothalamus (1.29), raphe nuclei (1.06), and locus coeruleus (1.00). The VMAT2 activity was slightly higher in brain stem (0.72), hippocampus (0.24), amygdala (0.47), and thalamus (0.18). All of the neocortical areas had low ^18^F-FP-(+)-DTBZ uptakes that were less than 5% of uptake in the anterior putamen. The VMAT2 density in cerebellum (10.60% SUR) was slightly higher than that in neocortex. In addition, the uptake value in anterior putamen was three-fold higher than those of cerebellum and neocortex. Furthermore, no significant correlation of regional SURs to age was found in subcortical regions, but with a trend of decline. To make the data publicly available, Table S1 showed the regional SURs from each subject in this study.

**Table 1:** Regional mean VMAT2 uptake.

	Regions	Average left and right sides	SUR Normalize to Anterior Putamen(%)	Left side	Right side	p value^a^
***Subcortex***	Caudate	2.27±0.43	81.50	2.22±0.44	2.31±0.43	0.4812
	Anterior Putamen	2.78±0.48	100.00	2.79±0.45	2.77±0.51	0.5973
	Posterior Putamen	2.68±0.48	96.46	2.72±0.54	2.64±0.44	0.8053
	Substantia Nigra	1.08±0.25	38.75	1.09±0.27	1.06±0.23	0.7336
	Nucleus Accumbens	1.43±0.59	51.41	1.41±0.53	1.45±0.68	0.9626
	Raphe nuclei	1.06±0.18	37.94			
	Hippocampus	0.24±0.07	8.47	0.25±0.08	0.23±±0.07	0.4449
	Amygdala	0.47±0.13	16.84	0.48±0.13	0.46±0.13	0.8694
	Hypothalamus	1.29±0.20	46.47	1.30±0.22	1.28±0.21	0.7599
	Thalamus	0.18±0.09	6.30	0.18±0.09	0.17±0.09	0.9065
	Locus coeruleus	1.00±0.14	35.90			
	Brain Stem	0.72±0.13	25.96			
***Cerebellum***	Vermis	0.21±0.09	7.51			
	Cerebellum Cortex	0.29±0.09	10.60	0.27±0.09	0.32±0.08	0.1126
***Cingulate***	Anterior Cingulum	0.11±0.10	3.78	0.11±0.11	0.10±0.11	0.7600
	Middle Cingulum	0.11±0.08	4.05	0.12±0.08	0.11±0.09	0.6294
	Posterior Cingulum	0.07±0.10	2.40	0.07±0.11	0.06±0.10	0.4174
***Neocortex***	Superior Frontal	0.09±0.05	3.10	0.09±0.05	0.09±0.06	0.9812
	Middle Frontal	0.08±0.05	3.01	0.08±0.05	0.09±0.06	0.8780
	Inferior Frontal	0.01±0.05	0.39	0.01±0.05	0.01±0.05	0.7240
	Medial Frontal	0.07±0.06	2.63	0.07±0.06	0.08±0.07	0.5250
	Motor Area	0.01±0.05	0.32	0.00±0.06	0.02±0.05	0.5883
	Superior Parietal	−0.04±0.06	−1.48	−0.02±0.06	−0.06±0.06	0.0651
	Inferior Parietal	0.01±0.04	0.36	0.01±0.04	0.01±0.04	0.8782
	Superior Temporal	0.00±0.06	0.00	−0.04±0.07	−0.03±0.08	0.6892
	Middle Temporal	0.08±0.05	2.84	0.08±0.05	0.08±0.05	0.8687
	Inferior Temporal	0.08±0.03	2.99	0.08±0.03	0.08±0.04	0.5545
	Superior Occipital	−0.03±0.04	−1.19	−0.04±0.04	−0.02±0.04	0.0896
	Middle Occipital	0.01±0.03	0.53	0.01±0.03	0.02±0.04	0.8408
	Inferior Occipital	-	-	0.01±0.03	−0.01±0.03	0.0706

a difference between left and right side.

The regional mean SURs were divided into 4 groups of subcortex, cerebellum, cingulate, and neocortex. Percentage SURs normalized to that of anterior putamen and P-value of bilateral difference were also shown.

**Figure 2:**
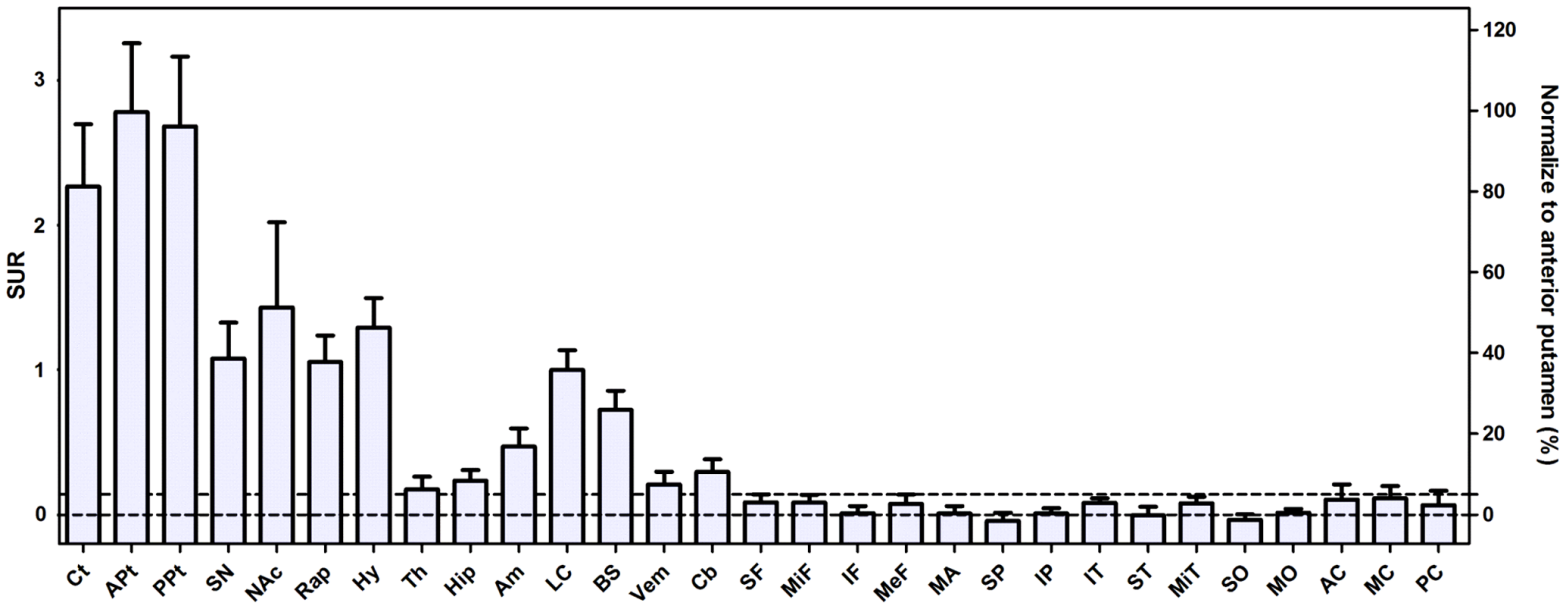
The mean regional SUR value and percentage SUR normalized to that of anterior putamen. Two dash lines indicated levels of 0% (lower) and 5% (upper) SUR to the anterior putamen, respectively. This clearly illustrated the distribution spectrum of VMAT2 density in the regions of subcortex, cerebellum, cingulate and neocortex. Ct: Caudate, APt: Anterior Putamen, PPt: Posterior Putamen, SN: Substantia nigra, NAc: Nucleus accumbens, Rap: Raphe, Hy: Hypothalamus, Th: Thalamus, Hip: Hippocampus, Am: Amygdala, LC: Locus coeruleus, BS: Brain stem, Vem: Vermis, Cb: cerebellum cortex, SF: Superior Frontal, MiF: Middle Frontal, IF: Inferior Frontal, MeF: Medial Frontal, MA: Motor Area, Olf: Olfactory, SP: Superior Parietal, IP: Inferior Parietal, ST: Superior Temporal, MiT: Middle Temporal, IT: Inferior Temporal, SO: Superior Occipital, MO: Middle Occipital, AC: Anterior Cingulate, MC: Middle Cingulate, PC: Posterior Cingulate.

Figure 2 displays the mean SUR value and percentage of regional SURs normalized to that of anterior putamen. According to the main types of monoaminergic axons/neurons and functional pathways in these brain areas, the regional SURs could be further divided into several parts: the nigrostriatal (DA), mesolimbic (DA), NE, SE and mixed pathways. Caudate and putamen innervated by DA neurons of SN had the highest SURs. In addition to the nigrostriatal pathway, NAc, the major relay nucleus of mesolimbic system receiving the DA projections from ventral tegmentum area (VTA), had the second highest SUR of the whole brain (51.4%). Hypothalamus innervated by both serotonin and norepinephrine axons also had high VMAT2 density (46.5%). Moreover, the VMAT2 uptake in locus coeruleus innervating pure norepinephrine axons was slightly lower than hypothalamus (35.9%). In the serotonin system, the highest uptake of ^18^F-FP-(+)-DTBZ was found in the raphe nucleus (37.9%). In the limbic system, the binding of ^18^F-FP-(+)-DTBZ was the highest in amygdala followed by hippocampus with SURs of 16.8% and 8.5%, respectively. The uptakes of ^18^F-FP-(+)-DTBZ were much lower in all cortical regions (<5%). The SUR of cerebellar cortex was higher than those of neocortex (10.6%).

## Discussion

The present study provided the distribution of VMAT2 in healthy aging brains by 2D and 3D ^18^F-FP-(+)DTBZ PET imaging (Figure 1). The brain structures with high VMAT2 density, including striatum, SN, raphe nuclei, NAc and limbic system, were clearly illustrated by ^18^F-FP-(+)-DTBZ PET imaging. In particular, the 3D images of ^18^F-FP-(+)-DTBZ PET demonstrated the anatomical relationship more precisely than the 2D images between the brain structures. The 3D images could help physicians and researchers to recognize the different VMAT2 distribution in individual case. With this, the usefulness of ^18^F-FP-(+)-DTBZ in detecting the disease-related alteration of VMAT2 density by visual assessment should be expected. Ultimately, the diagnostic accuracy in many neuropsychiatric disorders, such as PD and depression could be improved.

Recently, Tong et al. showed VMAT2 protein distribution in autopsied normal human brain by quantitative immunoblotting [12]. They concluded that the VMAT2 protein was the highest in putamen, followed by caudate, SN, hypothalamus and red nucleus. The cerebellar and cerebral cortices contain negligible VMAT2 protein versus the striatum. In the present study, the quantification analysis showed that the VMAT2 level was the highest in the putamen and caudate (the nigrostriatal pathway), followed by NAc (mesolimbic system) and hypothalamus. Low uptakes of ^18^F-FP-(+)-DTBZ were observed in cortex and cerebellum. Our results showed that the ratios of regional SURs normalized to that of the anterior putamen displayed similar pattern to the expression of VMAT2 protein obtained from autopsied human brains [12], [19]. Furthermore, the SURs in SN and raphe nuclei were approximately 60%∼70% lower than that in putamen for normal brains. This finding was also similar to the postmortem study that the densities of VMAT2 in SN and midbrain were about 20%∼30% of that in putamen for healthy controls [12]. It is noted that the VMAT2 level in raphe nuclei is mainly due to serotonergic innervation. In addition, as compared to the SUR in caudate, higher ^18^F-FP-(+)-DTBZ binding was observed in both anterior and posterior putamen but with no statistically significant difference (p = 0.6220) between these two putaminal VOIs in our study. The different VMAT2 distribution between caudate and putamen observed in the autopsied result [12], as well as previous in vivo ^18^F-FP-(+)-DTBZ and ^11^C-DTBZ studies [4], [5], [20], suggested a slightly lower density of VMAT2 in caudate than in putamen. Similar result of insignificant difference was found in the ^11^C-DTBZ study but with slightly lower uptake level in anterior putamen than posterior putamen [20]. Similar to the data in a recent report [14], our study also showed a relatively lower VMAT2 density in the NAc as compared to both caudate and putamen in normal brains.

The degeneration of dopamine neurons is not only confined to the nigrostriatal pathway for patients with early PD. For example, a recent FDOPA study reported additional degeneration in motor and anterior cingulate cortices [21]. Therefore, the capability of quantification for VMAT2 level in the extrastriatal regions might provide more information for studying non-motor symptoms in PD [21]. However, the previous in vivo and in vitro studies [12], [22], [23] reported low specific VMAT2 binding in the cortex. Another in vivo study also suggested that the cortical VMAT2 binding was too low to be reliably quantified using (+)-stereoisomer of ^11^C-DTBZ [20]. Consistently, our study indicated lower neocortical VMAT2 density (<5% SUR of anterior putamen) and thus being challenging for further quantification study in these regions. Nevertheless, many regions of the monoaminergic system, including NAc, amygdala, hypothalamus, raphe, and even thalamus and hippocampus, contain sufficient VMAT2 density to be quantifiable. Our results showed that ^18^F-FP-(+)-DTBZ PET imaging could detect the monoaminergic integrity in these extrastriatal regions. It suggested that ^18^F-FP-(+)-DTBZ PET might be a useful imaging biomarkers, not only for PD, but also for other neuropsychiatric disorders, such as affective disorders and schizophrenia.

The measurement of the ^18^F-FP-(+)-DTBZ uptake in this study was based on a semiquantitation method [5], [24]. The usual standard uptake value ratio (SUVR) was measured as an alternative estimate of the distribution volume ratio (DVR) which indicates distribution ratio of total ligand binding to the displaceable ligand amount [25]. Then SUR (specific uptake ratio = SUVR-1.0) was calculated as an estimate for amount of specific binding in brain imaging [18], [26] to provide a more informative interpretation of the VMAT2 binding. Here, occipital cortex was used as the reference region since it was found with very low VMAT2 expression as reported by in vitro studies [12], [19]. Some earlier studies applied cerebellum as a reference region but more consistent and higher binding values were observed when occipital region was used as the reference [27]. Similar to previous studies [20], [27], [28], our study also displayed a relatively higher SUR in the cerebellum and this might again explain why the lower binding values in the targets resulted from using cerebellum as the reference region. Therefore, the same distribution pattern but lower SUR values than those from the reference region of occipital cortex can be obtained when using cerebellum as the reference region. The measures based on using the occipital cortex as the reference region was reported with higher reliability than that from cerebellum in a test-retest study of ^11^C-DTBZ [27]. Other than occipital cortex and cerebellum, white matter might be considered as a reference region. However, heterogeneous distribution of free and nonspecific binding and difficulty in consistent region delineation for white matter are two main challenges [20]. Further study is necessary to investigate these effects for ^18^F-FP-(+)-DTBZ. Nevertheless, a similar amount of VMAT2 proteins in both cerebellum and occipital was reported from an in vitro study [12]. The discrepancy that imaging study showed relatively higher in vivo VMAT2 binding in cerebellum as compared to occipital might be due to protein availability between in vivo and in vitro, and possible partial volume effect in PET imaging [12] caused by the relatively small size of cortical regions. For the later reason, future work should include partial volume correction to improve the quantitation and to study this discrepancy.

Our study showed no significant aging-related decline in the nigrostriatal system, brain stem, hypothalamus, amygdala and hippocampus. The result of no significant correlation of VMAT2 density in the nigrostriatal pathway with age change was consistent with the in vitro [12], [29] and in vivo PET studies [27], [30]. On the contrary, the age-related decline of dopamine transporters (DAT) is well documented [26], [31], [32], [33], [34]. In addition, some in vivo VMAT2 studies using ^11^C-DTBZ also reported normal nigrostriatal aging effect [31], [35]. Our study suggested that VMAT2 was a relatively stable target during aging process. Age might not be an important confounding factor in the interpretation of ^18^F-FP-(+)-DTBZ PET images. Therefore, unlike DAT imaging, to “compare age-matched controls” may not be so critical for the ^18^F-FP-(+)-DTBZ PET studies for diseases involving the nigrostriatal system, at least for the population aged more than 50 years [26]. However, the aging effect of the ^18^F-FP-(+)-DTBZ binding in a younger population cannot be overlooked and needs further study.

Three-dimensional volume visualization has already demonstrated improvement in clinical care and diagnosis power for many imaging applications [36], [37], [38]. Due to the availability of a PET scanner with higher resolution and sensitivity, our study displayed a clear 3D visualization of in vivo VMAT2 binding in various brain structures. The use of 3D visualization for VMAT2 distribution might add additional diagnostic power in VMAT2 imaging studies, and deserves further studies to explore potential diagnostic applications. Future work should also include a test-retest reliability analysis before applying this tracer for use in general clinical diagnosis.

## Conclusion

This work provided 2D and 3D imaging features of the *in vivo* VMAT2 distribution in the healthy aging human brains using ^18^F-FP-(+)-DTBZ PET imaging. The VMAT2 distribution pattern was not only explored in the usual nigrostriatal pathways, but also the mesolimbic and mesocortical pathways, serotonin and norepinephrine pathways. The *in vivo* imaging characteristics of VMAT2 displayed similar distribution patterns to the *in vitro* VMAT2 protein levels reported in a recent autopsy study. We concluded that 3D visualization and higher image quality of ^18^F-FP-(+)-DTBZ PET imaging can improve diagnosis power for VMAT2 studies. The capability of ^18^F-FP-(+)-DTBZ PET in detecting VMAT2 of subcortical regions may help in clinical evaluation of many neuropsychiatric disorders that involved these monoaminergic systems.

## Supporting Information

Figure S1 **The 3D views of the average VMAT2 distribution in normal aging brain using ^18^F-FP(+)-DTBZ.** This illustrates the 3D visualization of subcortical VMAT2 binding in different angles. The image was displayed and overlaied on a corresponding CT image.
(MPG)

Table S1 **Regional SUR for each subject.** The regional SURs from each subject were listed and divided into 4 groups of subcortex, cerebellum, cingulate, and neocortex. SUR from bilateral regions were averaged. Please see Fig. 2 for acronyms used in this table.
(DOCX)
