# Supplementary material for: Brain Imaging of Vesicular Monoamine Transporter Type 2 in Healthy Aging Subjects by 18F-FP-(+)-DTBZ PET
Source: PLoS One. 2013 Sep 30;8(9):e75952. doi: 10.1371/journal.pone.0075952 (PMC3786914; doi:10.1371/journal.pone.0075952)
Supplement: Table S1 — Regional SUR for each subject. The regional SURs from each subject were listed and divided into 4 groups of subcortex, cerebellum, cingulate, and neocortex. SUR from bilateral regions were averaged. Please see Fig. 2 for acronyms used in this table. (DOCX) [file pone.0075952.s002.docx]

**Table S1. Regional SUR for each subject**

| **Subjects** | **Ct** | **APt** | **PPt** | **SN** | **NAc** | **Rap** | **Hy** | **Th** | **Hip** | **Am** | **Vem** | **Cb** | **SF** | **MiF** | **IF** | **MeF** | **IP** | **MiT** | **SO** | **MO** | **AC** | **MC** | **PC** |
| --- | --- | --- | --- | --- | --- | --- | --- | --- | --- | --- | --- | --- | --- | --- | --- | --- | --- | --- | --- | --- | --- | --- | --- |
| **S0001** | 2.52 | 3.17 | 3.11 | 1.32 | 1.41 | 1.13 | 1.47 | 0.20 | 0.20 | 0.44 | 0.21 | 0.40 | 0.07 | 0.07 | 0.02 | 0.08 | 0.00 | 0.05 | -0.01 | -0.02 | 0.03 | 0.09 | 0.01 |
| **S0002** | 2.19 | 2.94 | 2.50 | 1.02 | 1.90 | 0.92 | 1.18 | 0.11 | 0.27 | 0.48 | 0.16 | 0.43 | 0.06 | 0.07 | 0.06 | 0.08 | -0.01 | 0.06 | -0.03 | 0.02 | 0.07 | 0.11 | 0.02 |
| **S0003** | 2.40 | 2.85 | 2.70 | 1.19 | 2.36 | 1.17 | 1.22 | 0.08 | 0.29 | 0.71 | 0.09 | 0.30 | 0.08 | 0.10 | 0.04 | 0.07 | 0.04 | 0.10 | 0.02 | 0.03 | 0.13 | 0.15 | 0.10 |
| **S0004** | 2.23 | 2.81 | 2.44 | 0.85 | 1.11 | 0.85 | 1.07 | 0.17 | 0.21 | 0.29 | 0.23 | 0.28 | 0.04 | 0.03 | -0.03 | 0.01 | -0.03 | 0.05 | -0.01 | 0.04 | 0.07 | 0.08 | 0.11 |
| **S0005** | 1.72 | 2.14 | 2.24 | 0.81 | 0.83 | 0.69 | 1.12 | 0.20 | 0.21 | 0.26 | 0.27 | 0.28 | 0.10 | 0.11 | 0.05 | 0.09 | 0.02 | 0.06 | -0.02 | 0.02 | 0.11 | 0.10 | 0.08 |
| **S0006** | 1.95 | 2.52 | 2.63 | 0.94 | 0.49 | 0.94 | 1.11 | 0.11 | 0.18 | 0.25 | 0.22 | 0.39 | 0.03 | 0.01 | -0.01 | 0.00 | -0.01 | 0.07 | 0.01 | 0.05 | -0.03 | 0.03 | 0.06 |
| **S0007** | 1.78 | 2.27 | 2.23 | 0.81 | 1.08 | 0.87 | 1.02 | 0.07 | 0.11 | 0.37 | 0.28 | 0.32 | 0.08 | 0.06 | -0.10 | 0.02 | 0.05 | 0.01 | 0.00 | 0.04 | -0.15 | -0.10 | 0.01 |
| **S0008** | 1.74 | 2.26 | 2.30 | 0.66 | 1.01 | 0.85 | 1.08 | 0.30 | 0.11 | 0.31 | 0.25 | 0.25 | 0.04 | 0.06 | -0.06 | 0.03 | 0.02 | 0.08 | -0.04 | 0.00 | 0.02 | 0.04 | -0.07 |
| **S0009** | 2.66 | 3.54 | 3.02 | 1.40 | 2.82 | 1.22 | 1.47 | 0.27 | 0.32 | 0.67 | 0.23 | 0.36 | 0.11 | 0.13 | 0.08 | 0.12 | 0.08 | 0.08 | 0.03 | 0.07 | 0.19 | 0.17 | 0.03 |
| **S0010** | 2.69 | 2.97 | 3.25 | 1.05 | 1.45 | 1.05 | 1.49 | 0.12 | 0.21 | 0.44 | 0.34 | 0.43 | 0.06 | 0.05 | 0.02 | 0.05 | 0.04 | 0.06 | -0.05 | 0.01 | 0.09 | 0.14 | 0.05 |
| **S0011** | 3.29 | 4.08 | 3.85 | 1.69 | 2.25 | 1.54 | 1.86 | 0.32 | 0.34 | 0.65 | 0.29 | 0.25 | 0.11 | 0.08 | 0.04 | 0.10 | 0.03 | 0.11 | 0.00 | 0.03 | 0.15 | 0.12 | -0.09 |
| **S0012** | 2.63 | 3.20 | 3.29 | 1.13 | 1.40 | 0.95 | 1.50 | 0.15 | 0.24 | 0.56 | 0.16 | 0.28 | 0.06 | 0.06 | 0.00 | 0.06 | -0.08 | 0.07 | -0.08 | -0.01 | 0.11 | 0.10 | 0.09 |
| **S0013** | 2.37 | 2.88 | 2.62 | 1.29 | 0.66 | 0.94 | 1.18 | 0.13 | 0.28 | 0.42 | 0.15 | 0.25 | 0.06 | 0.08 | 0.01 | 0.07 | -0.02 | 0.06 | -0.04 | -0.01 | 0.11 | 0.12 | 0.11 |
| **S0014** | 2.29 | 2.56 | 2.12 | 0.95 | 1.09 | 1.07 | 1.26 | 0.18 | 0.23 | 0.49 | 0.14 | 0.31 | 0.05 | 0.05 | -0.01 | 0.04 | -0.01 | 0.07 | -0.05 | 0.03 | 0.11 | 0.10 | 0.04 |
| **S0015** | 1.28 | 2.06 | 1.57 | 0.69 | 1.41 | 1.18 | 1.12 | -0.05 | 0.10 | 0.40 | 0.00 | 0.24 | 0.02 | 0.03 | -0.04 | -0.01 | -0.01 | 0.03 | -0.09 | 0.02 | 0.01 | 0.02 | -0.12 |
| **S0016** | 2.33 | 2.85 | 2.75 | 1.18 | 2.13 | 1.10 | 1.36 | 0.10 | 0.30 | 0.49 | 0.29 | 0.35 | 0.09 | 0.05 | -0.04 | 0.05 | -0.01 | 0.07 | -0.05 | 0.00 | 0.02 | 0.04 | 0.05 |
| **S0017** | 2.43 | 3.25 | 3.17 | 1.17 | 1.00 | 0.94 | 1.59 | 0.18 | 0.34 | 0.49 | 0.30 | 0.38 | 0.05 | 0.06 | 0.01 | 0.06 | 0.02 | 0.04 | -0.06 | 0.01 | 0.11 | 0.12 | 0.06 |
| **S0018** | 2.38 | 2.53 | 2.68 | 1.27 | 0.97 | 1.18 | 1.32 | 0.19 | 0.23 | 0.46 | 0.28 | 0.26 | 0.08 | 0.09 | -0.02 | 0.05 | -0.03 | 0.08 | -0.02 | 0.02 | 0.14 | 0.14 | 0.06 |
| **S0019** | 1.83 | 2.50 | 2.60 | 0.97 | 1.07 | 1.23 | 1.27 | 0.24 | 0.15 | 0.53 | 0.23 | 0.25 | 0.12 | 0.09 | 0.02 | 0.09 | 0.04 | 0.10 | -0.03 | 0.01 | 0.21 | 0.25 | 0.20 |
| **S0020** | 2.10 | 2.57 | 2.59 | 1.16 | 1.75 | 1.17 | 1.30 | 0.24 | 0.32 | 0.60 | 0.20 | 0.25 | 0.25 | 0.25 | 0.10 | 0.28 | 0.03 | 0.20 | -0.13 | -0.05 | 0.31 | 0.23 | 0.24 |
| **S0021** | 2.55 | 2.80 | 2.90 | 1.24 | 1.76 | 1.19 | 1.26 | 0.28 | 0.28 | 0.52 | 0.24 | 0.18 | 0.21 | 0.20 | 0.09 | 0.20 | 0.06 | 0.19 | -0.03 | 0.01 | 0.29 | 0.30 | 0.30 |
| **S0022** | 2.52 | 2.47 | 2.48 | 0.92 | 1.53 | 1.01 | 1.18 | 0.25 | 0.25 | 0.48 | 0.03 | 0.05 | 0.12 | 0.11 | 0.00 | 0.09 | -0.01 | 0.11 | -0.06 | 0.00 | 0.22 | 0.15 | 0.13 |
